# Can graphene make better HgCdTe infrared detectors?

**DOI:** 10.1186/1556-276X-6-250

**Published:** 2011-03-23

**Authors:** Wen Xu, Youpin Gong, Liwei Liu, Hua Qin, Yanli Shi

**Affiliations:** 1 Department of Physics, Yunnan University, Kunming 650091, China; 2 Key Laboratory of Materials Physics, Institute of Solid State Physics, Chinese Academy of Sciences, Hefei 230031, China; 3 Suzhou Institute of Nano-tech and Nano-bionics, Chinese Academy of Sciences, Suzhou, China; 4 Kunming Institute of Physics, Kunming, China

## Abstract

We develop a simple and low-cost technique based on chemical vapor deposition from which large-size graphene films with 5-10 graphene layers can be produced reliably and the graphene films can be transferred easily onto HgCdTe (MCT) thin wafers at room temperature. The proposed technique does not cause any thermal and mechanical damages to the MCT wafers. It is found that the averaged light transmittance of the graphene film on MCT thin wafer is about 80% in the mid-infrared bandwidth at room temperature and 77 K. Moreover, we find that the electrical conductance of the graphene film on the MCT substrate is about 25 times larger than that of the MCT substrate at room temperature and 77 K. These experimental findings suggest that, from a physics point of view, graphene can be utilized as transparent electrodes as a replacement for metal electrodes while producing better and cheaper MCT infrared detectors.

## Introduction

As an ideal two-dimensional (2D) electronic system (2DES), graphene (single or a few layers of carbon atoms arranged in a hexagonal lattice) [1] has excellent electronic, electrical transport, and optical properties and interesting physical features [2]. Electronically, the carrier density in graphene [3] can be as high as 10^13 ^cm^-2^. It is much larger than that in conventional III-V and SiGe-based 2DESs. More importantly, the carrier density in graphene can be turned easily and efficiently through applying the gate voltages [4]. From an electrical transport point of view, graphene has very high carrier mobility [5] which can reach up to 20 m^2^/Vs at room temperature. This value of carrier mobility is about 100 times larger than that in conventional Si-based materials. Furthermore and optically, graphene has a very high light transmittance across the spectrum from the UV to the infrared. The light transmission coefficient for monolayer or bilayer graphene on SiO_2 _or Si substrates is about 98 or 96%, respectively, in the visible regime [6]. To utilize all these excellent properties and important features for device applications, one of the most significant and practical applications for graphene is in the area of transparent conducting material for optoelectronic devices such as photodetectors and optical displays. Recently, graphene has been proposed as a replacement for the conventional indium tin oxide (ITO) transparent electrodes in producing better and cheaper LCD devices [7]. Such an important application of graphene is based mainly on its excellent electrical, transport, and optical properties in the visible bandwidth. In this study, we would like to explore the possibility to apply graphene in the infrared optoelectronic devices such as infrared photodetectors and light sources.

## Principle of designing graphene transparent electrodes

From a physics point of view, two basic requirements have to be satisfied when a material is selected to be used as transparent electrodes. The selected material must have both high electrical conductance and high light transmittance on the substrate. We found recently [8] that the electrical conductance *σ *of graphene on a dielectric substrate depends on dielectric constant *κ *of the substrate material according to the following equation:(1)

where *σ*_0 _= *e*^2^*/h*, *n*_e _is the areal electron density in graphene, *n*_I _is the charged impurity concentration in graphene, and *E*_F _is the Fermi energy (or chemical potential) in graphene. This equation implies that graphene has a better electrical conductance on a substrate with a larger dielectric constant. The physics reason behind this effect is that the main scattering mechanism to determine the carrier mobility in graphene layer is the charged impurity scattering within a relatively wide temperature range [8], especially for relatively low-density sample systems [8,9]. In an air- graphene-substrate system in which the graphene layer is undoped and the system is unbiased, the charged impurities arise mainly from the substrate, and the electron density in the system is relatively low. Meanwhile, because graphene is a very thin layer of 2D carbon crystal, graphene can surely be of high light transmittance. It is known that for an air-graphene-substrate system, the transmission coefficient of the graphene layer can be calculated according to the following equatuion [10](2)

where ϵ _1_= 1 for air, ϵ_2 _is the high-frequency dielectric constant of the substrate material, and *σ*(*ω*) is the optical conductance of graphene. This equation suggests that the light transmittance of the graphene layer on a substrate decreases with the increasing dielectric constant of the wafer material at a fixed *σ*(*ω*). Moreover, it was found, both experimentally [11] and theoretically [12,13], that in the short wavelength or visible regime *σ*(*ω*) = *Ne*^2^*/*4*ħ *is a universal optical conductance with *N *being the number of layers in graphene film, whereas in the mid-infrared (MIR) bandwidth, there is an optical absorption window existing in graphene. The MIR absorption window in graphene is induced by inter- and intra-band optical absorption channels required for different transition energies [12,13] and, therefore, the width and depth of the absorption window depend sensitively on carrier density (or gate voltage) [11] and temperature [12,13]. The presence of the optical absorption window in the MIR bandwidth indicates that graphene has an even better light transmittance in the infrared regime. Hence, graphene can be applied for MIR optical and optoelectronic devices, especially for infrared transparent conducting material for various applications.

## MCT infrared detectors

On the other hand, HgCdTe (MCT)-based infrared detectors are popularly used as high-quality night-vision devices for MIR detection [14]. The MCT infrared detectors are made mainly from photoconductors, photodiodes, and avalanche photodiodes [14] in which electrodes are required to be made. Because the conventional ITO materials have relatively poor light transmittance in the MIR bandwidth, metal electrodes are often used for making MCT infrared detectors [14]. Normally, the metal electrodes cover about 20-30% area in the active regime of the MCT chips (see, e.g., Figs. 13 to 15 in [14]). If the metal electrodes in the MCT infrared detectors are replaced by the transparent ones, then the radiation area becomes enlarged and, hence, we are able to enhance the efficiency of the MIR detection and to improve the quality of the infrared images. In this study, we demonstrate that graphene is a good candidate for transparent electrodes to be applied for the production of MCT infrared detectors.

## Sample preparation

**Figure 1:**
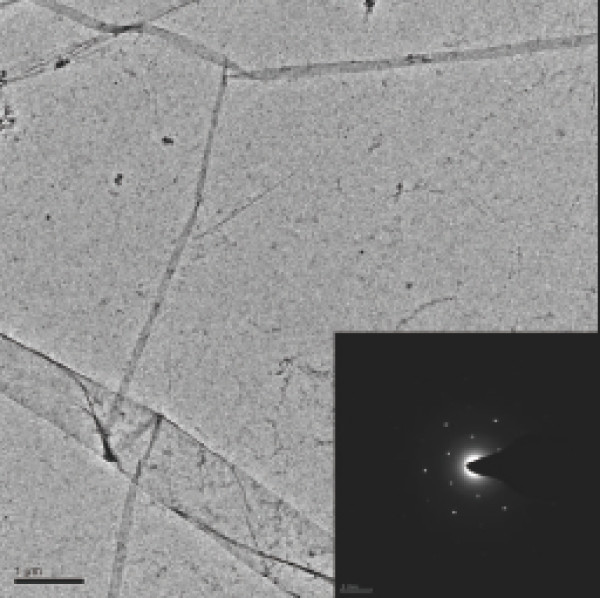
**TEM image of the graphene film grown by the CVD technique**. The inset is the SAED pattern of the graphene film.

In this study, the graphene films are grown using the standard chemical vapor deposition (CVD) technique. CH_4 _is taken as carbon precursor flowing over a 500-nm-thick Ni film catalyst on a SiO_2 _substrate. The reaction temperature is 900°C, and the flow rates of CH_4 _and H_2 _are about 50 and 150 sccm, respectively. The reaction time is around 5 min. In this way, we can produce reliably the large-size and high-quality graphene films with a fewer layers (5-10) of graphene. This is verified by the measurements of optical transmittance and transmission electron microscopy (TEM). Figure 1 shows the low-resolution TEM image and the SAED pattern of the graphene film, wherein we can see that a highly crystallized structure of few-layer graphene film has been achieved with the typical sixfold symmetry. Using this technique, the size of the graphene film produced is mainly determined by the size of the Ni film which plays a role as catalyst. The graphene layer on Ni film is then transferred onto the thin MCT wafers at room temperature through (i) spin casting with PMMA at 3000 rpm/min for 1 min; (ii) baking at 170°C for 2 h; (iii) peeling off graphene on Ni film by etching in 1 mol/l NaOH at 80°C, followed by etching underlying Ni film by FeCl_3 _solution; and (iv) transferring the graphene film onto MCT wafer in water at room temperature. In addition to the large size of the graphene film that can be transferred onto the MCT wafer, another advantage of this technique is that there is not at all a thermal or mechanical damage to the MCT wafer samples. We know that Hg in MCT evaporates at about 180°C. Thus, the conventional method used for growing graphene film on substrate, such as MBE growth and thermal expansion, cannot be used for growing graphene directly on the MCT substrates. The MCT wafers used in this investigation are with the approximate thicknesses of 1 *μ*m and the sizes of 1 cm^2^. The MCT wafers are placed on sapphire substrate.

## Experimental results

**Figure 2:**
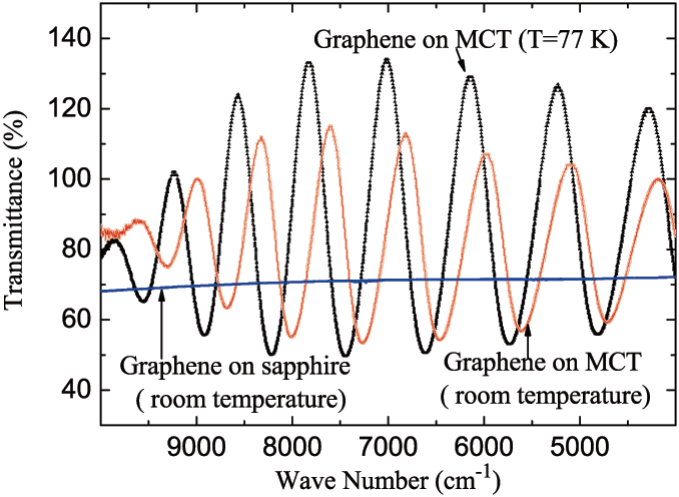
**Infrared transmission spectrum for 5-10 layers of graphene on thin MCT wafer at room temperature and 77 K**. The transmittance for graphene film on sapphire at room temperature is shown as a reference.

In Figure 2, we can see infrared transmission spectrum for 5-10-layer graphene film on MCT thin wafer at room temperature and 77 K. The transmission spectrum for graphene film on sapphire is also presented at room temperature as a reference. The transmission coefficient for 5-10 layers of graphene on sapphire is about 70% in the MIR regime at room temperature. This confirms further that the film consists of approximately 5-10 graphene layers. The oscillations of the transmittance for graphene films on MCT wafers are induced because the thickness of the MCT wafer (*L*_MCT_) is approximately of the radiation wavelength *λ*. Here the transmission coefficient of the graphene film *T*_G _is deducted from the total transmittance *T*_t _of the air-graphene-substrate system, and the transmittance of the MCT wafer alone *T*_MCT _from *T*_g _= *T*_t_*/T*_MCT_. We note that such a formula holds only for a case where the thicknesses of two material layers are much larger than *λ*. There is no simple and analytic formula to deduct the transmittance of a thin film (*L*_1 _≪ *λ*) from the total transmittance and the transmittance of the wafer (*L*_2 _~ *λ*). Because, in this case, the thickness of the graphene film is much smaller than *λ *and *L*_MCT _~ *λ*, *T*_G _may be larger than that in the oscillation regime as shown in Figure 2. When the graphene film with 5-10 alyers is placed on a thin MCT wafer, the geometric mean of the envelope curve of *T*_G _is nearly 80% in the mid-IR spectrum at room temperature and 77 K. The stronger oscillations of *T*_G _can be observed at 77 K. These results indicate that a graphene film with 5-10 graphene layers can have nearly 80% light transmittance when it is placed on top of a thin MCT wafer.

**Figure 3:**
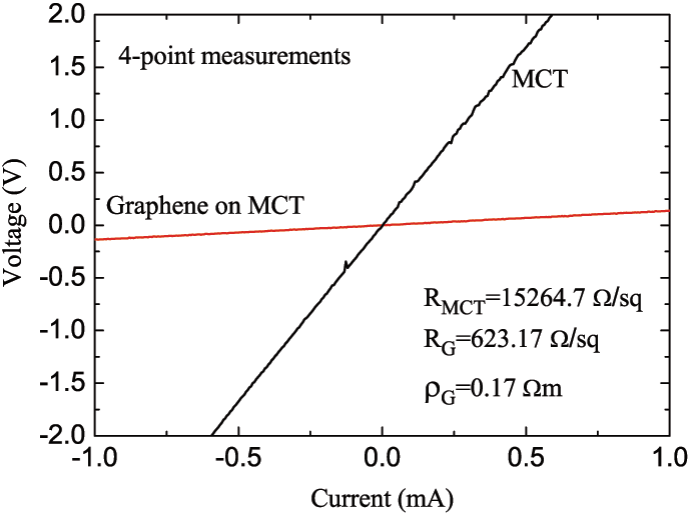
***I*-*V *relations, obtained from four-point measurements, for graphene on MCT substrate and for sole MCT wafer at room temperature**. Here, *R*_MCT _and *R*_G _are the resistances, respectively, for the MCT substrate and for the graphene film, and _G _is the resistivity for graphene layer on MCT thin wafer.

The *I*-*V *relations obtained from the four-point measurements for graphene film on MCT substrate and for MCT wafer alone are shown in Figure 3 at room temperature. We find that the resistance of the graphene film on MCT (*R*_G _~ 623 Ω/sq) is nearly 25 times less than that of the MCT wafer (*R*_MCT _~ 15265 Ω/sq). The resistivity of the graphene film with 5-10 graphene layers on MCT wafer is about 0.17 Ω·*m *at room temperature. There is a small difference between *R*_MCT _and *R*_G _values at *T *= 77 K and at room-temperature. We have also measured the photo-resistance of the graphene film on the thin MCT substrate when MIR radiation fields, polarized linearly, are applied. It is found that there is a slight decrease in both *R*_G _and *R*_MCT _in the presence of MIR radiation. Those experimental findings indicate that the conductance and photo-conductance of a graphene film with 5-10 graphene layers are much lager than those of the MCT thin wafer in the MIR bandwidth at room temperature and 77 K (the operating temperature of the MCT infrared detectors).

HgCdTe or MCT is a material with relatively high dielectric constant (*κ *~ 14). This is one of the reasons why relatively high electric conductance can be achieved for graphene film on the MCT substrate. For 5-10 layers of graphene film on the MCT wafer, the light transmittance in the MIR bandwidth is about 80%. This is slightly lower than that on the SiO_2_/Si substrate in the visible regime. We find that the graphene films can be placed nicely on the MCT thin wafers with smooth surface. No crack or folding of the graphene film is found in our samples.

## Conclusions

In this study, we have developed a simple and low-cost technique to grow graphene films reliably and to transfer the graphene films easily onto the thin HgCdTe wafers at room temperature. This technique can produce large-size graphene films and does not cause thermal and mechanical damages to the MCT thin wafer. We have found that multi-layer (e.g., 5-10 layers) graphene films on MCT thin wafer can have high light transmittance in the MIR bandwidth and relatively high electrical conductance at room temperature and 77 K. The most important conclusion that we drew from this study is that the light transmittance (about 80% in the MIR bandwidth) and the electrical conductance (about 25 times larger than that in the wafer itself at room temperature and 77 K) of the graphene film on the MCT thin wafer can meet nicely the requirements for the infrared transparent electrodes. These interesting findings allow us to propose that graphene can be used as a replacement for metal electrodes to produce better and cheaper MCT infrared detectors. Graphene has been proposed as a replacement for the ITO as transparent electrodes for optical devices such as LCD and LED [7]. The results and analyses presented in this article indicate that graphene has even better features to be utilized as infrared transparent-conducting materials. This study can be considered as a stimulus for future applications of graphene in infrared optoelectronics and infrared optical devices.

## Abbreviations

2D: two-dimensional; 2DES: 2D electronic system; CVD: chemical vapor deposition; MIR: mid-infrared; ITO: tin oxide; TEM: transmission electron microscopy.

## Competing interests

The authors declare that they have no competing interests.

## Authors' contributions

WX proposed the research work, coordinated the collaborations, and carried out theoretical study and analyzes of the experimental results. YPG, LWL and HQ participated in the growth of graphene samples, in the transformation of graphene films onto the MCT wafers and in the electrical and optical measurements of the study. YLS prepared the HgCdTe wafers used in this study. All authors read and approved the final manuscript.
